# Eosinophils and Chronic Respiratory Diseases in Hospitalized COVID-19 Patients

**DOI:** 10.3389/fimmu.2021.668074

**Published:** 2021-06-02

**Authors:** Marcela Valverde-Monge, José A. Cañas, Blanca Barroso, Diana Betancor, Laura Ortega-Martin, Alicia Gómez-López, María Jesús Rodríguez-Nieto, Ignacio Mahíllo-Fernández, Joaquín Sastre, Victoria Del Pozo

**Affiliations:** ^1^ Allergy Unit, Hospital Universitario Fundación Jiménez Díaz, Madrid, Spain; ^2^ Immunology Department, Instituto de Investigación Sanitaria Fundación Jiménez Díaz (IIS-FJD), Madrid, Spain; ^3^ CIBER de Enfermedades Respiratorias (CIBERES), Instituto de Salud Carlos III, Madrid, Spain; ^4^ Pulmonology Unit, Hospital Universitario Fundación Jiménez Díaz and Hospital General de Villalba, Madrid, Spain; ^5^ Epidemiology and Biostatistics Unit, Instituto de Investigación Sanitaria Fundación Jiménez Díaz (IIS-FJD), Madrid, Spain

**Keywords:** chronic respiratory diseases, COVID-19, eosinopenia, eosinophils, asthma, COPD (chronic obstructive pulmonary disease), OSA (obstructive sleep-apnea)

## Abstract

**Background:**

Studies on the role of eosinophils in coronavirus disease 2019 (COVID-19) are scarce, though available findings suggest a possible association with disease severity. Our study analyzes the relationship between eosinophils and COVID-19, with a focus on disease severity and patients with underlying chronic respiratory diseases.

**Methods:**

We performed a retrospective analysis of 3018 subjects attended at two public hospitals in Madrid (Spain) with PCR-confirmed SARS-CoV-2 infection from January 31 to April 17, 2020. Patients with eosinophil counts less than 0.02×10^9^/L were considered to have eosinopenia. Individuals with chronic respiratory diseases (n=384) were classified according to their particular underlying condition, i.e., asthma, chronic pulmonary obstructive disease, or obstructive sleep apnea.

**Results:**

Of the 3018 patients enrolled, 479 were excluded because of lack of information at the time of admission. Of 2539 subjects assessed, 1396 patients presented an eosinophil count performed on admission, revealing eosinopenia in 376 cases (26.93%). Eosinopenia on admission was associated with a higher risk of intensive care unit (ICU) or respiratory intensive care unit (RICU) admission (OR:2.21; 95%CI:1.42-3.45; *p*<0.001) but no increased risk of mortality (*p*>0.05).

**Conclusions:**

Eosinopenia on admission conferred a higher risk of severe disease (requiring ICU/RICU care), but was not associated with increased mortality. In patients with chronic respiratory diseases who develop COVID-19, age seems to be the main risk factor for progression to severe disease or death.

## Introduction

In December 2019, a new human coronavirus, officially denominated severe acute respiratory syndrome coronavirus 2 (SARS-CoV-2), was identified in Wuhan, China. On March 11 2020, the World Health Organization declared the illness caused by the novel virus - coronavirus disease 2019 (COVID-19) – to have reached pandemic levels (1). COVID-19 is a predominantly respiratory disease, with an extremely variable presentation ranging from minimal flu-like symptoms to significant hypoxia and acute respiratory distress syndrome. However, it can also affect other organs and systems (2). All age groups are at risk for infection, although older adults (> 65 years) are more vulnerable, with coronary artery disease, diabetes, and hypertension identified as risk factors for mortality on univariable analysis (3).

As respiratory viruses are the most common triggers of asthma exacerbation, there is widespread concern that SARS-CoV-2 may pose a particular threat to asthma patients (4). However, coronaviruses are not as strongly linked to asthma exacerbations (4) as are others, with rhinoviruses constituting the greatest risk (5). In a previous publication, we reported the prevalence and characteristics of patients with asthma in a small cohort of hospitalized COVID-19 patients, finding an asthma prevalence of 5.80%, a rate closely resembling that observed in the overall Spanish population (6). Our previous findings (6) are supported by a recent study on asthma and COVID-19, in which the authors found no clear evidence that patients with asthma are at a higher risk of infection or of developing severe illness (7). In a Spanish cohort of 545 patients with severe uncontrolled asthma under biologic treatment, Rial et al. (8) described a similar prevalence and severity of COVID-19 between these and non-severe asthma patients. However, this is not always the case for other chronic respiratory diseases. Available data clearly suggests a higher risk of severe COVID-19 for chronic obstructive pulmonary disease (COPD) patients, although the reported prevalence seems to be similar or even lower than that of the general population (9). In the CORONADO study, including diabetic patients admitted to hospital with COVID-19, Cariou et al. (10) reported that treated obstructive sleep apnea (OSA) was independently associated with an increased risk of mortality.

Eosinophils have an important role in the pathogenesis of varied respiratory conditions, including chronic respiratory diseases such as asthma and COPD. They represent a small (1-3%) percentage of all circulating leucocytes (11). As components of the immune system, eosinophils are involved in a profusion of homeostatic and inflammatory responses, and have been observed to take part in antiviral host defense (12, 13). Eosinophils recognize and react against viruses through indirect mechanisms such as pattern recognition receptors (toll-like receptors, nucleotide-binding oligomerization domain-like receptors, and he receptor for advanced glycation end products), high mobility group box 1 (HMGB1) receptor expression, and participation in antigen presentation, and through direct mechanisms such as the release of preformed eosinophil-derived neurotoxin and eosinophil cationic protein and extracellular traps (14). Dysregulation of eosinophils contributes to many eosinophil-associated diseases as idiopathic hypereosinophilic syndrome, severe eosinophilic asthma, chronic rhinosinusitis with nasal polyposis (CRSwNP), eosinophilic esophagitis, eosinophilic granulomatosis with polyangiitis, bullous pemphigoid, and atopic dermatitis (15). Under certain conditions, eosinophils migrate to target tissues such as the respiratory tract, where the presence of degranulated eosinophils in lung tissue is associated with the pathogenesis of bronchial smooth muscle hyperreactivity (16). In both allergic and severe non-atopic late-onset asthma, eosinophilia has been correlated with disease severity, frequent exacerbations and tissue remodeling (17). In COPD, higher blood eosinophil counts may In predict a positive response to corticosteroid treatment (18), although recently they have also been associated with higher readmission rates for severe exacerbations (17). Regarding COVID-19, eosinopenia has been observed in 51% of hospitalized patients (19). This finding is associated with longer hospital stays (20), while normalization of the blood eosinophil (EOS) count may be indicative of recovery (21). In contrast, in a larger cohort, Lucas et al. (22) observed increased levels of eosinophils among patients with severe COVID-19. Their research on the immune response in patients with moderate or severe COVID-19 demonstrates an increase in type 2 effectors (i.e., interleukin-5, interleukin-13, and immunoglobulin E) in addition to higher eosinophil counts. The immune process that causes eosinopenia in COVID-19 is still unclear and is probably multifactorial, as a recent review on the mechanism of action displayed by eosinophils against respiratory viruses (14) concludes.

In this study, our main objective was to determine the association of eosinophils with the severity of COVID-19, specifically in patients with chronic respiratory diseases (CRD).

## Materials and Methods

### Subjects and Data Acquisition

Patients attending either of two public hospitals in Madrid, Spain, (Hospital Universitario Fundación Jiménez Díaz and General Hospital of Collado Villalba) from January 31 to April 17, 2020 with polymerase chain reaction (PCR)-confirmed SARS-CoV-2 infection were included. The participating hospitals follow common clinical practice guidelines for admission and inpatient management of SARS-CoV-2 infection (described in **Supplementary Material**). Maintaining patient confidentially at all times, medical records were retrieved from a Microsoft SQL Server Integration Services (SSIS) reporting database and analyzed using R version 3.5.2 (R Project for Statistical Computing; R Foundation), MATLAB 9.7-R2019b (MathWorks Natick, MA, USA), and IBM SPSS software version 25 (IBM, Armonk, NY, USA). Approval was obtained from the institutional Ethics Committee. We retrospectively studied patient charts to obtain data on demographic characteristics, baseline comorbidities (hypertension, cardiac disease, cancer, renal and neurological conditions, and type 2 diabetes mellitus), laboratory test results on admission and discharge, smoking status, use of systemic corticosteroids during hospitalization, need for intensive care unit (ICU) or respiratory intermediate care unit (RICU) admission, and outcome on discharge (death or survival).

At the time of admission, the hospital computer system requires physicians to enter mandatory information on patients’ preexisting conditions. Electronic records of patients thus classified as presenting an underlying respiratory disease were further analyzed in detail and categorized as presenting asthma, COPD, OSA, and/or other chronic respiratory diseases (**Supplementary Material**). Eosinopenia was defined as an EOS count of less than 0.02 × 10^9^/L (19) with regards to data analysis.

### Statistics

For statistical analysis, we expressed categorical variables as frequencies and percentages, with comparisons made using Fisher’s exact test. Odds ratio (OR) with a 95% confidence interval (CI) was calculated for clinical variables to report association between the risk of death for different chronic respiratory diseases, and the risk of ICU admission and sex. Continuous variables were summarized as mean ± standard deviation (SD) or median and interquartile range (IQR).

Data were compared using an unpaired, two-tailed Mann-Whitney test for comparisons between two groups or the Kruskal-Wallis with Dunn *post hoc* test for multiple comparisons. For paired data, comparisons between two groups were performed using a two-tailed Wilcoxon matched-pairs test or repeated-measures ANOVA with Dunn *post hoc* test for multiple comparisons. The Kolmogorov-Smirnov test was used to evaluate the normal distribution of data. A p-value less than 0.05 was considered significant. Statistical analyses were performed using GraphPad Prism 8 (GraphPad Software Inc, San Diego, CA, USA).

Missing data were entered using multiple imputations, implemented using the MICE R package. In order to determine the most relevant variables for predicting mortality and ICU admission, univariable and multivariable logistic regression models were used. Univariable models were summarized by the OR, 95% CI, and p-value. Multivariable models were chosen by the best subset regression method, using the Akaike information criterion (AIC) to select the model with the best fit. Leave-one-out (LOO) cross-validation was carried out to evaluate the performance of the models in classification. This performance was summarized by the ROC curve and the area under the curve. Also, OR, 95% CI, and p-values of statistical significance of OR, and Hosmer-Lemeshow test were reported.

## Results

### Study Population

Of the initial 3018 patients enrolled, 479 were excluded because of lack of completion of the respiratory items of the initial physician-performed admission questionnaire. We divided the remaining 2539 patients into two major groups: those with (n = 384) and without (n = 2155) an underlying chronic respiratory disease. The prevalence of chronic respiratory diseases was 15.12%.

The non-chronic respiratory disease group (NCRD) was balanced in terms of gender (female: 51.04%), with a mean age of 61.09 ± 19.30 years, and included only 124 (5.75%) active smokers. ICU or RICU admission was required for 125 individuals (5.80%), and there were 234 total deaths (10.86%), mostly among males (58.12%), with this association being statistically significant (OR = 1.45, 95% CI = 1.13-1.85; *p*<0.01).

The CRD group was predominantly male (57.29%), with a mean age of 71.41 ± 14.83 years, and included 30 (7.8%) active smokers. 89 subjects (23.18%) had been previously diagnosed with COPD, 81 (21.09%) with OSA, 113 (29.42%) with asthma and 98 with other CRD such as bronchiectasis, interstitial lung disease (ILD), or chronic pulmonary thromboembolism. Only 24 patients (6.2%) required ICU/RICU admission (7 asthma, 10 OSA, 2 COPD, 4 other CRD; Table S1). Eighty-six deaths were recorded: 9 (10.46%) had asthma, 18 (20.93%) had OSA, 27 (31.39%) had COPD, and 32 (37.21%) had another CRD. The statistical analyses comparing CRD and NCRD are reported in **Table 1**.

**Table 1:** Demographic and clinical data of patients from the CRD and NCRD populations.

	NCRD population (n=2155)	CRD population (n=384)	p-value
***Demographic variables***			
Age, years (mean ± SD)	61.1 ± 19.3	71.4 ± 14.8	****
Female (%)	1100 (51.0)	164 (42.7)	****
BMI (mean ± SD)	27.0 ± 5.1	30.6 ± 26.2	***
Smoking status (%)			
Never	1675 (77.7)	217 (56.5)	****
Former smoker	356 (16.5)	137 (35.7)	****
Smoker	124 (5.8)	30 (7.8)	N.S.
***Hospitalization parameters***			
Fatal outcome (%)	234 (10.8)	86 (22.4)	****
ICU (%)	125 (5.8)	24 (6.2)	N.S.
First SpO2 (%)	94.0 (92.0-96.0)	94.0 (91.0-96.0)	N.S.
***Inflammatory pattern***			
Eosinophils (×10^9^/L)	0.07 (0.01-0.18)	0.11 (0.02-0.21)	**
Eosinopenia (%)	373 (17.3)	73 (19.0)	N.S.
Leukocytes (×10^9^/L)	7.45 (5.14-10.27)	6.90 (5.46-8.80)	N.S.
Lymphocytes (× 10^9^/L)	1.40 (0.9-2.00)	1.60 (0.90-2.30)	N.S.
Basophils (×10^10^/L)	0.34 ± 0.5	0.43 ± 0.5	*
Neutrophils (× 10^9^/L)	4.10 (3.10-6.00)	4.40 (3.05-5.80)	N.S.
Monocytes (×10^9^/L)	0.50 (0.30-0.60)	0.50 (0.40-0.70)	N.S.
***Laboratory Parameters***			
D-dimer (μg/mL)	0.52 (0.29-0.97)	0.48 (0.27-1.09)	N.S.
Ferritin (μg/mL)	606 (254–1609)	156 (69.7-357.0)	****
C-reactive protein (μg/mL)	4.11 (1.10-8.99)	2.80 (0.83-8.43)	N.S.
***Comorbidities***			
Heart disease (%)	371 (17.22)	162 (42.19)	****
Diabetes mellitus (%)	317 (14.71)	86 (22.40)	***
Renal disease (%)	141 (6.54)	58 (15.10)	****
Neurological disease (%)	214 (9.93)	70 (18.23)	****
Cancer (%)	108 (5.01)	38 (9.89)	***
High blood pressure (%)	837 (38.84)	217 (56.51)	****

CRD, chronic respiratory disease group; NCRD, non-chronic respiratory disease group; BMI, body mass index; ICU, intensive care unit; N.S., no statistically significant difference found. *p < 0.05; **p < 0.01; ***p < 0.001; ****p < 0.0001.

A comparison of NCRD and CRD patients revealed that individuals with NCRD were significantly younger (*p*<0.0001). CRD patients were predominantly male and presented higher mortality rates and higher eosinophil and basophil blood counts on admission. The association between CRD and other comorbidities was stronger than in the NCRD group, particularly for hypertension and cardiac disease (**Table 1**).

### CRD Subgroup Analysis

Asthma patients had a mean age of 62.28 ± 18.34 years; though COPD and OSA mostly affected males, most subjects in the asthma group were female (**Table 2**). Seven asthma patients (5.26%) required ICU/RICU admission, of whom six were discharged from the hospital, and one died. A closer look at the nine deaths (7.96%) in the asthma group reveals that most of them took place in patients over 82 years of age with underlying cardiac diseases and hypertension. On statistical analysis, asthma patients showed a lower risk of death due to COVID-19 when compared to patients with COPD (OR = 0.20, 95% CI = 0.09-0.45; *p*<0.0001), OSA (OR = 0.30, 95% CI = 0.13-0.72; *p*<0.01) and the other-CRD group (OR = 0.19, 95% CI = 0.09-0.44; *p*< 0.0001).

**Table 2:** Demographic and clinical data of patients with asthma, COPD and OSA.

	Asthma (n=113)	COPD (n=89)	OSA (n=81)	Asthma *vs* COPD	Asthma *vs* OSA	COPD *vs* OSA
***Demographics***						
Age, years (mean ± SD)	62.3 ± 18.3	76.2 ± 10.0	68.6 ± 11.5	***		**
Female (%)	66 (58.4)	19 (21.3)	20 (24.7)	****	****	
BMI (mean ± SD)	29.3 ± 9.0	28.0 ± 6.7	33.7 ± 7.5		***	***
Smoking status (%)						
Never	83 (73.4)	18 (20.2)	42 (51.8)	****	**	****
Former smoker	27 (23.9)	57 (64.0)	34 (42.0)	****	*	**
Smoker	3 (2.7)	14 (15.7)	5 (6.2)	**		
***Inflammatory pattern***						
Eosinopenia (%)						
*Previous*	3 (2.7)	2 (2.2)	1 (1.2)			
*Admission*	8 (7.1)	12 (13.5)	13 (16.0)			
*Discharge*	24 (21.2)	19 (21.3)	21 (25.9)			
Leukocytes (×10^9^/L)						
*Previous*	6.70 (5.53-8.15)	7.16 (5.68-8.65)	7.19 (5.94-8.61)			
*Admission*	6.41 (5.61-8.59)	7.84 (5.60-8.89)	6.93 (5.10-8.50)			
*Discharge*	7.18 (5.23-9.06)	7.55 (5.50-10.38)	8.54 (6.24-10.24)			
Lymphocytes (×10^9^/L)						
*Previous*	1.60 (0.90-2.38)	1.20 (0.70-1.95)	1.80 (1.13-2.60)			
* Admission*	1.00 (0.80-1.55)	0.70 (0.40-1.10)	1.10 (0.60-1.60)	**		*
* Discharge*	0.00 (0.00-0.10)	0.00 (0.00-0.10)	0.00 (0.00-0.10)	*		*
Basophils (×10^10^/L)						
* Admission*	0.42 ± 0.5	0.41 ± 0.53	0.45 ± 0.5			
* Discharge*	0.19 ± 0.4	0.18 ± 0.39	0.26 ± 0.61			
Neutrophils (×10^9^/L)						
* Admission*	4.35 (3.00-5.78)	5.10 (3.98-6.65)	4.30 (2.90-5.48)			*
Monocytes (×10^9^/L)						
* Admission*	0.55 (0.40-0.70)	0.60 (0.30-0.80)	0.50 (0.40-0.70)			
***Laboratory parameters***						
D-dimer (μg/mL)						
* Admission*	0.43 (0.18-0.67)	0.59 (0.40-1.63)	0.65 (0.33-1.27)			
Ferritin (μg/mL)						
* Admission*	168 (76.7-721)	152 (101-315)	169 (74.5-452)			
* Discharge*	907 (352-1336)	539 (271-1632)	642 (434-982)			
C-reactive protein (μg/mL)						
* Admission*	1.16 (0.49-4.83)	3.08 (0.53-11.67)	3.10 (0.50-7.96)			
***Hospitalization parameters***						
Exitus (%)	9 (8.0)	27 (30.3)	18 (22.2)	****	**	
ICU (%)	7 (5.3)	2 (2.2)	10 (12.3)			*
First SpO2 (%)	94.0 (92.0-96.0)	94.0 (91.0-96.0)	94.0 (91.0-96.0)			
***Comorbidities***						
Cardiovascular (%)	33 (29.2)	45 (50.6)	30 (37.0)	**		
Diabetes mellitus (%)	17 (15.0)	23 (25.8)	24 (29.6)		*	
Renal (%)	13 (13.3)	14 (15.7)	9 (11.1)			
Neurological (%)	15 (13.3)	18 (20.2)	13 (16.0)			
Cancer (%)	7 (6.2)	11 (12.4)	8 (9.9)			
High blood pressure (%)	46 (40.7)	59 (66.3)	52 (64.2)	***	**	

Comparisons were performed between chronic respiratory diseases subgroups. COPD, chronic respiratory disease; OSA, obstructive sleep apnea; BMI, body mass index; ICU, intensive care unit. *p < 0.05; **p < 0.01; ***p < 0.001; ****p < 0.0001.

Patients in the COPD group had a mean age of 76.19 ± 9.98 years and were predominantly male. The majority of smokers were included in this group (**Table 2**). The overall prevalence of COPD was 3.51% in this cohort. We also observed a high percentage of COPD patients with cardiac disease and hypertension. Of the 27 deaths recorded, the majority occurred in patients over 70 years of age with a variety of comorbidities such as cardiac diseases, renal diseases, and hypertension. Unfortunately, the two patients who required ICU/RICU admission both died.

Patients with OSA had a mean age of 68.62 ± 11.50 years, were mainly male, obese, and presented cardiac diseases and hypertension (**Table 2**). Of note, four of the 10 OSA patients requiring ICU/RICU admission died, of which none presented cardiovascular comorbidities. A total of 18 OSA patients died,14 of whom were over 72 years old.

Upon statistical analysis of the three groups, we found that COPD patients were significantly older than those in the other groups (**Table 2**). OSA patients had a significantly higher body mass index (BMI), with majority of patients in this group suffering from obesity. Also, we observed a majority of females and non-smokers in the asthma group, a statistically significant difference with regards to the other groups. Regarding white blood cell count on admission and discharge, neither eosinophils nor monocytes showed significant differences between CRD subgroups. However, COPD patients had a lower lymphocyte count than that of OSA patients (*p*<0.05) and asthma patients (*p*<0.01) on discharge. Blood levels of D-dimer, ferritin or C-reactive protein showed no statistical difference among our three subgroups (**Table 2**). Overall, COPD patients had the highest mortality rate, and the results of a univariable logistic regression model showed that age was the main variable influencing death in the asthma (OR = 1.10, 95% CI = 1.03-1.17; *p*<0.01) and OSA group (OR = 1.08, 95% CI = 1.02-1.15; *p*<0.01). Using a multivariable logistic regression model, age again showed the highest association with mortality, regardless of other comorbidities or other independent variables (**Figure S1**).

### Eosinophil Behavior in Hospitalized COVID-19 Patients

**Figure 1:**
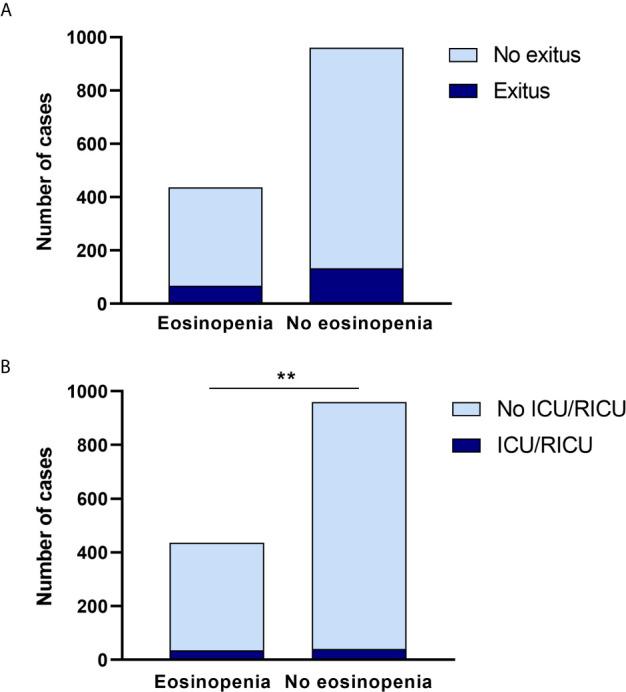
Eosinopenia can predict ICU/IRCU admission. **(A)** Eosinopenia on admission did not change significantly in regards to observed mortality in either group. **(B)** When the need for ICU/RICU admission was assessed, patients with eosinopenia on admission had a significantly higher risk in comparison to patients with no eosinopenia on admission. **p < 0.01.

Of the 2539 subjects assessed, we obtained an EOS count on admission for 1396. Considering eosinopenia as an EOS count of less than 0.02 ×10^9^/L, we found that 376 subjects had eosinopenia (26.93%). The group with eosinopenia was predominantly male (53.21%), 41 subjects (10.90%) required ICU/RICU admission, and 68 died (18.09%). The other 1020 patients belonged to the normal (non-eosinopenia) EOS group which was balanced in terms of gender (females 50.93%); 43 subjects (4.22%) were admitted to ICU/RICU and 134 died (13.14%). Statistical analysis suggests that eosinopenia on admission places patients at a higher risk of ICU/RICU admission (**Figure 1A**) but does not confer a higher risk of mortality (**Figure 1B**).

**Figure 2:**
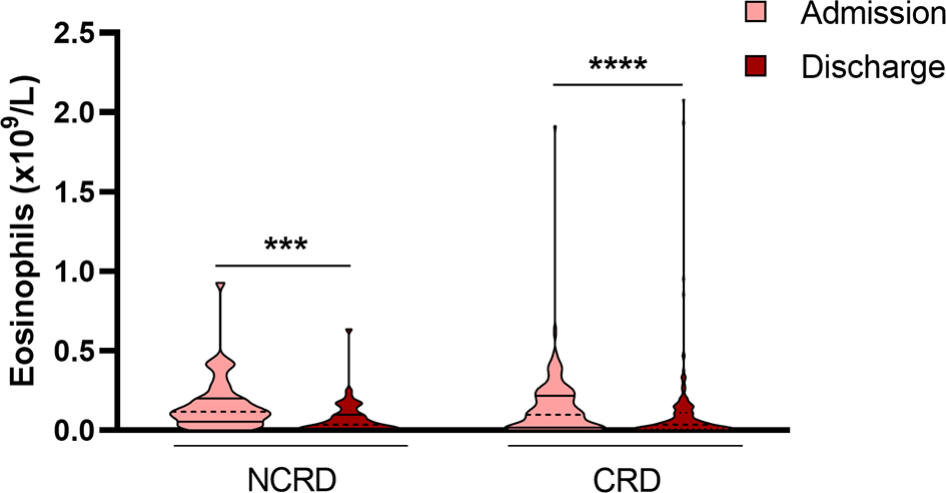
Patients at discharge show a lower number of blood eosinophils. Data revealed that eosinophils were higher on admission than at discharge in both groups (CRD and NCRD). Patients treated with systemic corticosteroids during hospitalization were excluded from analysis. ***p < 0.001; ****p < 0.0001.

When we analyzed EOS on admission, we found that patients in the CRD group had higher EOS counts than NCRD patients (**Figure 2**); this difference reached statistical significance (1.7 (0.3-3.1) *vs* 1.1 (0.2-2.7) eosinophils ×10^9^/L; *p*<0.01). We also compared EOS counts on admission to EOS counts at discharge in order to study the temporal evolution of this marker in both CRD and NCRD groups; of 2539 patients included, both values were available for only 627 (CRD=133 and NCRD=494). Subsequently, we excluded patients treated with systemic corticosteroids (394 subjects) as these drugs are known to deplete EOS and therefore act as a confounding factor; this left 233 patients for analysis (CRD=45 and NCRD=188). We observed that EOS counts were higher on admission than at discharge in both groups (**Figure 2**).

**Figure 3:**
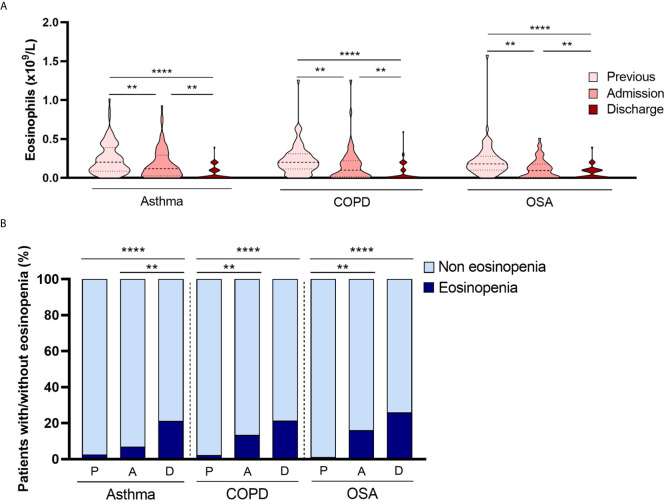
Eosinophil behavior among CRD subgroups. **(A)** EOS count showed a significant progressive decrease from admission to discharge in all CRD groups (asthma, COPD and OSA). **(B)** Patients with eosinopenia on admission were present in all CRD subgroups. Interestingly, in the asthmatic subgroup, patients with eosinopenia at discharge were significantly more numerous than at admission, a trend not observed in COPD and OSA patients. **p < 0.01; ****p < 0.0001.

As eosinophils are associated with chronic respiratory diseases, we also collected CRD patients’ EOS counts before hospital admission. We considered “eosinophils before admission” as the number of eosinophils from patients’ most recent complete blood count performed before testing positive for SARS-CoV2, an interval which varied from a month to 6 years. Data showed that eosinophils on admission were significantly lower than eosinophil counts before hospital admission. **Figure 3A** shows eosinophil data before admission, on admission and at discharge. The median EOS count before admission in asthma [0.21x10^9^/L (0.09-0.41)], COPD [0.20x10^9^/L (0.11-0.31)] and OSA [0.18x10^9^/L (0.10-0.28) were normal. Patients with eosinopenia on admission were present in all CRD subgroups (**Table 2**), but at discharge, the number of patients with eosinopenia increased (**Figure 3B**). These patterns of eosinophil behavior were observed across all CRD subgroups. We found no differences between CRD subgroups in terms of changes in eosinophil counts.

**Figure 4:**
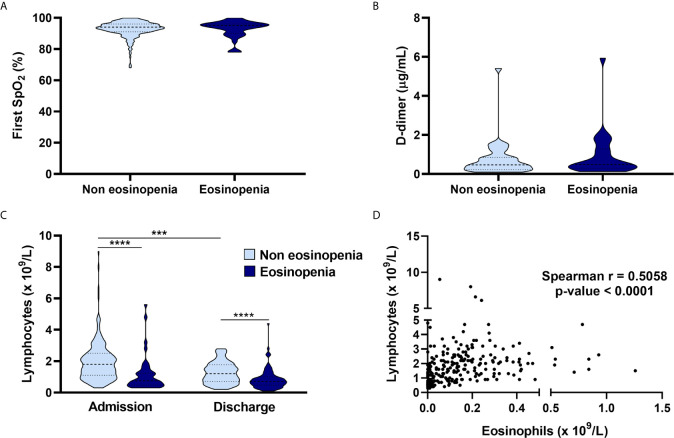
Relationship of eosinopenia with clinical and laboratory parameters. Comparison of the eosinopenia group and non-eosinopenia group regarding peripheral capillary oxygenation saturation on admission **(A)**, with no differences between groups; D-dimer on admission showed similar results **(B)**; the absolute lymphocyte count on admission was higher in the non-eosinopenia group, and both groups presented higher absolute lymphocyte counts on admission than at discharge **(C)**; suggesting a correlation between lymphocytes and eosinophils **(D)**. ***p < 0.001; ****p < 0.0001.

Peripheral capillary oxygen saturation (SpO_2_) and D-dimer values on admission showed no significant difference between the eosinopenia and non-eosinopenia groups (**Figures 4A, B**). Interestingly, the non-eosinopenia group had a significantly higher lymphocyte count than the eosinopenia group (**Figure 4C**). Lymphocytes on admission were significantly higher than lymphocytes at discharge in both the eosinopenia and non-eosinopenia groups (**Figure 4C**), suggesting a correlation between lymphocyte and eosinophil counts (**Figure 4D**). Also, levels of C-reactive protein did not exhibit any significant difference (**Table 2**).

## Discussion

Our main objective was to describe the role played by eosinophils in the severity of COVID-19 and to clarify the relationship between eosinophils and CRD. Our analysis suggests that eosinopenia on admission could be related with severe COVID-19 disease, resulting in a higher risk of ICU/RICU admission. This finding supports the results published by Xie et al. (20) in which patients with eosinopenia had worse radiographic findings, longer hospital stays, and more severe disease. We obtained a lower prevalence of eosinopenia (26.93%) on admission than other published series (19, 20), possibly owing to our larger sample size and also because the study period coincided with peak pollen season in Madrid.

Classically, eosinophils have been considered as important immune cells in the pathogenesis of inflammatory processes such as parasitic helminth infections and allergic or pulmonary diseases like allergic and non-allergic asthma; more recently, they have been linked to the immune response conferring host protection linked both, by direct or indirect mechanisms against viruses, bacteria and yeasts (23). The finding of eosinopenia on admission in hospitalized patients associated with worst recovery outcomes could be a signal of an intense immune response environment against the pathogen causing the disease. In fact, persistent peripheral eosinopenia has been established as a marker of poor outcomes in severe acute infections as bacterial sepsis (24). In regards to respiratory diseases, in patients with COPD exacerbations who need hospitalization, eosinopenia on admission has been associated with higher mortality and lengthier hospital stay than patients with normal eosinophils (25); nowadays, eosinopenia is included in an algorithm that help determine the prognosis of patients with acute exacerbation of COPD (26) on admission. It is also interesting to mention that eosinophil degranulation has been observed to be significantly decreased in the eosinophils from older subjects (50-88 y.o.) in comparison to younger subjects (20-40 y.o.) in patients with asthma (27); this finding may influence worst prognosis in hospitalized patients with infections in older patients. Human rhinovirus and respiratory syncytial rhinovirus (RSV) have been identified as major pathogens of virus-induced asthma exacerbations (28, 29); in fact, RSV infection in childhood places adults at risk of developing asthma (30) relationship to asthma and eosinophils, a few studies have demonstrated the relationship between eosinophils and asthma exacerbations. Calhoun et al. found the significant migration of eosinophils to lung tissue after the exposure to allergens in subjects with allergic rhinitis with previous infection due to rhinovirus in comparison with normal volunteers (31). Green et al. demonstrated that changes in controller medication of patients with asthma based on the count of eosinophils in sputum, led to a significant reduction in asthma exacerbations and in hospitalization admission due to asthma exacerbation (32) in comparison by management based on guidelines suggestion of symptoms and lung function tests; reinforcing the leading role of eosinophils in asthma exacerbations. Moreover, blood eosinophil counts over 400 cell/μL have been associated with a greater rate of asthma exacerbation and lower odds of achieving asthma control (33).

Our findings support those of a recent cluster analysis of a cohort of 825 hospitalized subjects with asthma, in which patients in cluster 3 (with older age, high daily inhaled corticosteroid dose, more intense maintenance medications, and severe or life-threatening exacerbations) characterized by a lower mean of blood eosinophils on admission (0.02x10^9^/L) presented the worst in-hospital prognosis with extended hospital stay and a higher need of ICU admission in comparison with the other two clusters (34).

Surprisingly, when analyzing eosinophil counts from admission to discharge, we found more patients with eosinopenia at discharge than on admission, both in the CRD and NCRD groups. For this analysis, patients who received systemic corticosteroids during their hospital stay were excluded, because this could act as a confounding factor. This finding contrasts with the rate of EOS recovery at discharge found by Xie G et al. (20). This discrepancy between the two reports may be the result of the larger sample size used in our study, or the fact that the complete EOS recovery observed by Xie et al. occurred approximately 18 days after disease onset. With regard to this last factor, our laboratory findings may reflect an earlier hospital discharge, as to alleviate the great strain on the healthcare system caused by the pandemic, COVID-19 patients in the Madrid region were discharged to an intermediate care medical facility before complete resolution of the disease. We found a positive correlation between lymphocytes and eosinophils, but did not observe a significant relationship between eosinopenia and D-dimer, SpO_2_ nor with C-reactive protein.

A recently published article analyzing immune response using peripheral blood from 253 patients hospitalized with COVID-19 shows that patients with severe COVID-19 tend to have increased eosinophils compared to those with moderate disease (22). However, as the aforementioned study quantified low density eosinophils and used a different technique, no accurate comparison can be made.

Our results show that eosinopenia on admission could be used to identify those patients at higher risk for severe COVID-19, defined as requiring ICU/RICU care.

In addition, we analyzed the differences in COVID-19 severity among hospitalized patients who had asthma, COPD, or OSA. These patients have higher mortality, possibly due to the high rate of older patients in the COPD group as our univariate and multivariate analysis results suggested (**Table S2** and **Figure S1**). It has been hypothesized that this greater severity is related to the higher expression of angiotensin-converting enzyme II (ACE-2) in the lower airways of active smokers with COPD, as this is the receptor that SARS-CoV-2 binds to and uses to enter epithelial cells (35). Also, the higher percentage of comorbidities associated in this group might play an important role where mortality is concerned.

The prevalence of patients with OSA was 3.19%. These patients seem to have a higher risk of requiring ICU/RICU admission than patients with COPD. This difference is likely related to the group’s younger age range and fewer associated comorbidities, which makes these patients more eligible to receive intensive care than the older, more comorbid patients with a lower chance of recovery in the COPD group. However, when OSA patients were compared to asthma patients, the two subgroups were found to have a similar mean age; statistical analysis does suggest a higher risk of mortality among OSA patients, which probably predicts a higher severity of COVID-19. Our findings are in accordance with a recent analysis by Maas et al. (36), who conclude that OSA patients were more prone to COVID-19 infection and at least double the risk of developing respiratory failure. On the other hand, obesity (defined as BMI ≥ 30.0 kg/m^2^) has been recognized as a risk factor for severe COVID-19; furthermore, obesity has been shown to increase the risk of hospital admission, ICU admission, and mortality in previous viral pandemics (37).

The prevalence of asthma patients in this study was 4.45%, with a predominantly female population, consistent with epidemiological observations of the non-COVID-19 asthma population (38). Our statistical analyses suggest that these patients have the same chance of severity, ICU/RICU care and mortality as NCRD patients. Based on their observations of lesser severity of COVID-19 in patients with a T2 phenotype asthma, Muñoz X. et al. (39) suggest that this phenotype could play a protective role against COVID-19. Extensive analyses have ruled out asthma as a risk factor for SARS-CoV2 infection or for severe COVID-19 (40).

High levels of ferritin and D-dimer on admission have been associated with severity of COVID-19 (41). Surprisingly, higher ferritin levels were found in the NCRD group than CRD patients even though a higher percentage of fatal outcome was observed in the CRD group. We suspect that this controversial result could be explained by the fact that NCRD was significantly younger than CRD; and as our multivariate analysis for the risk of mortality showed, age is the main risk factor. The laboratory parameters that have been associated with higher mortality rates are high levels of D-dimer and severe lymphopenia. The levels of C-reactive protein did not significantly differ between NCRD and CRD; nor between the CRD subgroups; this could be explained by the fact that in the context of viral pneumonia this biomarker tends to fall in the normal range and not be useful for determining prognosis (42). On comparison, none of our CRD subgroups showed any statistically significant differences in these three parameters.

In summary, although the immune mechanism of eosinopenia in COVID-19 remains unclear, patients with eosinopenia on admission have a higher risk of developing severe COVID-19 requiring ICU/RICU care, meaning that eosinopenia on admission can be used to predict the severity of the disease. Eosinophils are more likely to be low at discharge irrespective of severity. In our multivariate analysis to assess the risk of mortality in asthma, COPD and OSA patients, age was the main risk factor for mortality in patients with COVID-19.

## Data Availability Statement

The original contributions presented in the study are included in the article/**Supplementary Material**. Further inquiries can be directed to the corresponding author.

## Ethics Statement

The studies involving human participants were reviewed and approved by CEIC from University Hospital Fundación Jimenez Díaz. Written informed consent for participation was not required for this study in accordance with the national legislation and the institutional requirements.

## Author Contributions

MV-M and JAC were involved with the acquisition, analysis, and interpretation of data, manuscript drafting and critical revision for important intellectual content, the approval of the final version of the document. VP and JS were involved in the study concept and design, interpretation of data, critical revision of the manuscript for important intellectual content; study supervision, and approval of the final version of the manuscript. BB, DB, LO-M, AG-L, and MR-N were involved in data acquisition, critical revision of the manuscript for important intellectual content, and in the approval the final version of the document. IM-F was involved in the analysis and interpretation of data, critical revision of the manuscript for important intellectual content, and in the approval the final version of the manuscript. All authors contributed to the article and approved the submitted version.

## Conflict of Interest

JS reports having served as a consultant to Thermofisher, MEDA, Novartis, Sanofi, Leti, Faes Farma, Mundipharma, and GSK; having been paid lecture fees by Novartis, GSK, Stallergenes, Leti, and Faes Farma; as well as having received grant support for research from Thermofisher, Sanofi, and ALK. VP reports having served as a consultant to Astra Zeneca and GSK and having been paid lecture fees by both. MR-N reports having received grant support for research from Astra Zeneca and GSK, having served as a consultant to Astra Zeneca and GSK, and having received payments for lectures by both. JAC has received payment for lecture by Astra Zeneca. MV-M has received payment for lecture from GSK.

The remaining authors declare that the research was conducted in the absence of any commercial or financial relationships that could be construed as a potential conflict of interest.
