# Supplementary material for: Eosinophils and Chronic Respiratory Diseases in Hospitalized COVID-19 Patients
Source: Front Immunol. 2021 Jun 2;12:668074. doi: 10.3389/fimmu.2021.668074 (PMC8208034; doi:10.3389/fimmu.2021.668074)

**METHODS**

Guidelines for diagnosis, management and treatment for every COVID-19 hospitalized patient.

At the emergency department, subjects with suspicion of COVID-19 had a polymerase chain reaction (PCR) testing for SARS-CoV-2 performed and baseline peripheral oxygen saturation (SaO_2_) taken. If they had a high suspicion of COVID-19 based on symptoms (because the result of PCR was available until 24hrs after performed), abnormal clinical auscultation and Sat.O_2_ <94%; complementary studies were ordered (chest x-ray and blood test with hemogram, renal function parameters, hepatic function parameters, D-dimer, ferritin, acute cardiac disease enzymes).

When pneumonia was observed at chest x-ray and patient had other comorbidities (age>65years, hypertension (HTA), Diabetes type 2 (DM2), COPD, cancer, chronic cardiac disease, chronic liver disease and/or immunosuppression) and/or altered laboratory parameters (lymphopenia<800 cell count, neutrophilia>7500 cell count, ferritin over 500μg/mL, over LDH 350UI/L, over D-dimer 3000μg/mL, PCR over 10mg/dL) hospital admission was decided.

All hospitalized patients received treatment with lopinavir 200mg/ritonavir 50mg two pills every 12hrs for 7 days. Also, if patients did not suffer from hypertension nor from chronic kidney failure; treatment with cyclosporine A was offered and if agreed by the patient, an informed consent was signed by both patient and physician. Cyclosporine A was initiated based on weight (<60kg: 50mg-0-50mg; between 60-80kg: 100mg-0-50mg; over 80kg: 100mg-0-100mg); the dose was augmented every 48hrs to reach 5mg/kg/day and treatment was maintained during at least 15 days. All patients also received hydroxychloroquine 400mg every 12hrs for 5 days, low molecular weight heparin at prophylactic dose. If clinical deterioration occurred during hospitalization, intravenous methylprednisolone 250mg every day for 3 days and 80mg the next 2 days was administered.

**Other-CRD group**

The Other-CRD group was formed by 98 patients with different chronic respiratory diseases such as pulmonary carcinoma, chronic pulmonary thromboembolism, pneumothorax, solitary lung nodule, pulmonary sarcoidosis, bronchiectasis, pulmonary hypertension, tuberculosis residual lung damage, pneumatocele, diffuse interstitial pulmonary fibrosis, chronic pleural effusion, lung disease secondary to systemic erythematous lupus and some “under study” lung diseases without an established diagnosis. Demographic and clinical data of patients with Other-CRD are in Table S1.

**Table S1:** Demographic and clinical data of patients with Other-CRD.

| **Other-CRD Asthma COPD OSA**  **(n=98) vs vs vs**  **Other-CRD Other-CRD Other-CRD** | | | | |  |
| --- | --- | --- | --- | --- | --- |
| **Demographics**  Age, years (mean ± SD)  Female (%)  BMI (mean ± SD)  Smoking Status (%)  *Never*  *Former smoker*  *Smoker* | 76.59 ± 13.36 | ******* |  | ******* | |
|  | 49 (50.00) |  | ******** | ******* | |
|  | 27.42 ± 6.40 |  |  | ******* | |
|  |  |  |  |  | |
|  | 55 (56.12) | ****** | ******** |  | |
|  | 35 (35.71) |  |  |  | |
|  | 8 (8.16) |  |  |  | |
| **Inflammatory Pattern**  Eosinopenia (%)  *Previous*  *Admission*  *Discharge*  Leukocytes (x 10^9^/L)  *Previous*  *Admission*  *Discharge*  Lymphocytes (x 10^9^/L)  *Admission*  *Discharge*  Basophils (x 10^10^/L)  *Admission*  *Discharge*  Neutrophils (x 10^9^/L)  *Admission*  Monocytes (x 10^9^/L)  *Admission* |  |  |  |  | |
|  | 2 (2.04) |  |  |  | |
|  | 8 (8.16) |  |  |  | |
|  | 29 (29.59) |  |  |  | |
|  |  |  |  |  | |
|  | 6.76 (5.74-8.34) |  |  |  | |
|  | 6.45 (5.48-8.30) |  |  |  | |
|  | 7.59 (6.01-10.83) |  |  |  | |
|  |  |  |  |  | |
|  | 1.30 (0.83-2.20) |  | ******* |  | |
|  | 0.70 (0.50-0.70) |  |  |  | |
|  |  |  |  |  | |
|  | 0.42 ± 0.59 |  |  |  |  |
|  | 0.10 ± 0.29 |  |  |  |  |
|  |  |  |  |  |  |
|  | 4.15 (3.00-5.75) |  |  |  |  |
|  |  |  |  |  |  |
|  | 0.50 (0.30-0.70) |  |  |  |  |
| **Laboratory Parameters, median (IQR)**  D-dimer (μg/mL)  *Admission*  Ferritin (μg/mL)  *Admission*  *Discharge* |  |  |  |  | |
|  |  |  |  |  | |
|  | 0.63 (0.27-1.49) |  |  |  | |
|  |  |  |  |  |  |
|  | 102.00 (49.00-208.50) |  |  |  |  |
|  | 586.00 (311.50-1299.00) |  |  |  |  |
| **Hospitalization Parameters**  Exitus (%)  ICU (%)  First SaO2 (%) |  |  |  |  | |
|  | 30 (30.61) | ******** |  |  | |
|  | 5 (5.10) |  |  |  | |
|  | 94.00 (89.00-96.00) |  |  |  | |
| **Comorbidities**  Cardiovascular (%)  Diabetes (%)  Renal (%)  Neurological (%)  Cancer (%)  Hypertension (HTA) (%) |  |  |  |  | |
|  | 54/98 (55.10) | ******* |  | ***** | |
|  | 24/98 (24.49) |  |  |  | |
|  | 17/98 (17.35) |  |  |  | |
|  | 23/98 (23.47) |  |  |  | |
|  | 11/98 (11.22) |  |  |  | |
|  | 62/98 (63.27) | ****** |  |  | |

**Table S2: Univariate Model for CRD Subgroups:** The univariable logistic regression model where the dependable variable was a fatal outcome and the independent variables were age, sex, BMI and comorbidities; age was the main variable influencing death in the asthma, OSA and Other-CRD subgroups. **p*<0.05.

| **Subgroup** | **Variable** | **OR** | **(95%CI)** | **P** |
| --- | --- | --- | --- | --- |
| Asthma | Gender | 1.47 | (0.35, 6.19) | 0.603 |
|  | Age | 1.10 | (1.03, 1.17) | 0.005 |
|  | Heart Disease | 5.59 | (1.31, 23.9) | 0.022 |
|  | Diabetes Mellitus | 1.70 | (0.32, 8.95) | 0.535 |
|  | Renal Disease | 1.24 | (0.23, 6.79) | 0.803 |
|  | Neurological Disease | 1.21 | (0.26, 5.73) | 0.808 |
|  | Hypertension | 3.20 | (0.76, 13.5) | 0.117 |
|  | BMI | 1.03 | (0.94, 1.12) | 0.584 |
| COPD | Gender | 0.78 | (0.25, 2.43) | 0.669 |
|  | Age | 1.04 | (0.99, 1.09) | 0.086 |
|  | Heart Disease | 1.08 | (0.44, 2.66) | 0.873 |
|  | Diabetes Mellitus | 1.32 | (0.48, 3.62) | 0.592 |
|  | Renal Disease | 4.04 | (1.16, 14.1) | 0.042 |
|  | Neurological Disease | 3.99 | (0.65, 24.3) | 0.184 |
|  | Hypertension | 1.68 | (0.62, 4.59) | 0.311 |
|  | BMI | 0.98 | (0.89, 1.09) | 0.774 |
| OSA | Gender | 0.84 | (0.24, 2.92) | 0.784 |
|  | Age | 1.08 | (1.02, 1.15) | 0.008 |
|  | Heart Disease | 2.81 | (0.94, 8.42) | 0.068 |
|  | Diabetes Mellitus | 1.72 | (0.57, 5.17) | 0.335 |
|  | Renal Disease | 1.73 | (0.42, 7.14) | 0.456 |
|  | Neurological Disease | 2.88 | (0.90, 9.18) | 0.080 |
|  | Hypertension | 1.60 | (0.51, 5.05) | 0.426 |
|  | BMI | 0.95 | (0.86, 1.05) | 0.339 |
| Other-CRD | Gender | 1.47 | (0.62, 3.49) | 0.384 |
|  | Age | 1.07 | (1.02, 1.11) | 0.005 |
|  | Heart Disease | 2.00 | (0.82, 4.90) | 0.133 |
|  | Diabetes Mellitus | 1.51 | (0.57, 3.99) | 0.403 |
|  | Renal Disease | 0.86 | (0.21, 3.49) | 0.835 |
|  | Neurological Disease | 0.39 | (0.09, 1.75) | 0.254 |
|  | Hypertension | 2.44 | (0.92, 6.46) | 0.075 |
|  | BMI | 0.97 | (0.89, 1.06) | 0.508 |

***Figure S1:*** Different multivariable models were built, discarding the models with a Hosmer-Lemeshow p-value<0.01. Based on the area under the curve (AUC), none of the models was capable of reaching a significant capacity of predicting mortality. COPD, chronic obstructive pulmonary disease; OSA, obstructive sleep apnea; CRD, chronic respiratory diseases; BMI, body mass index.


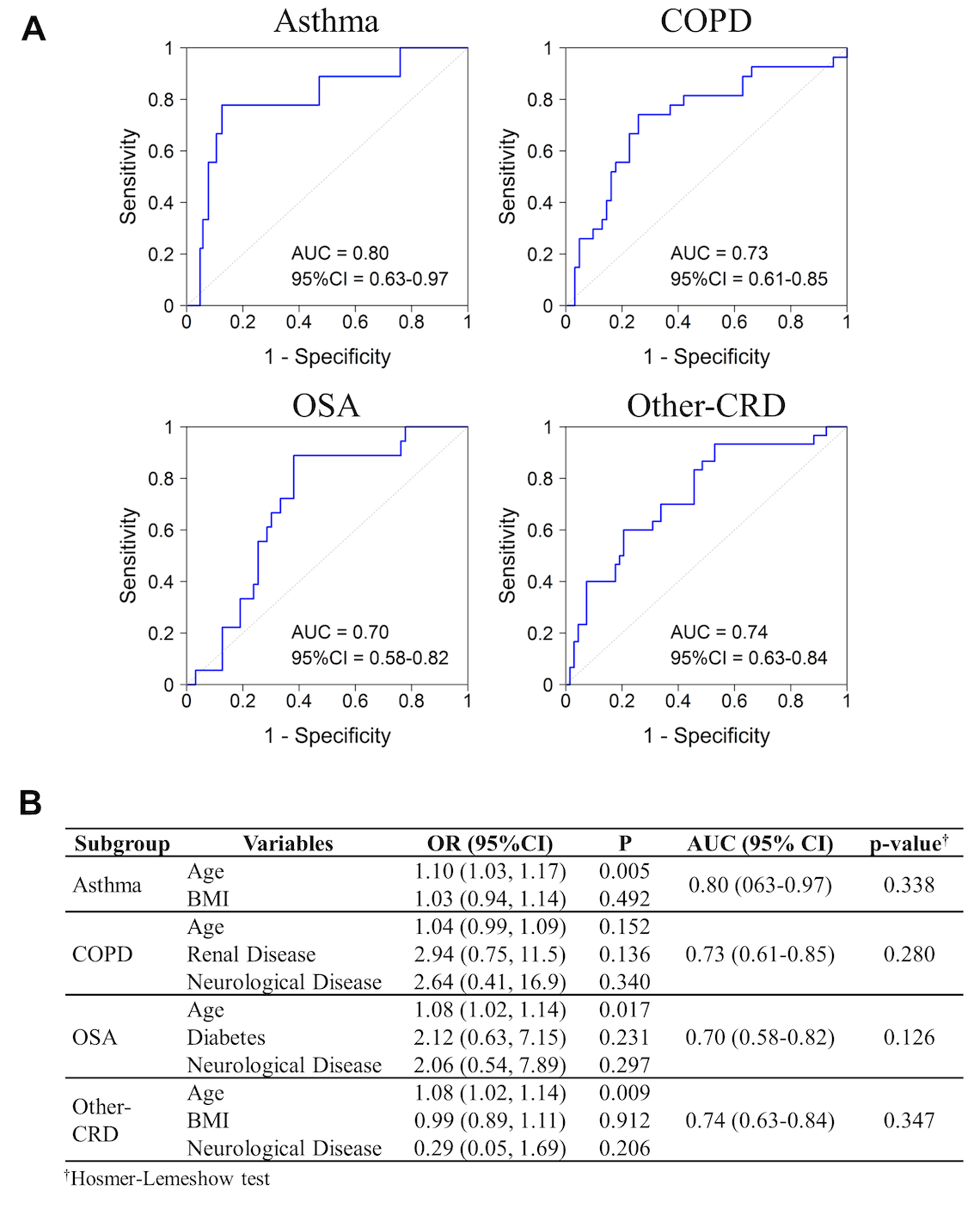

Supplement: Supplementary file 1 [file DataSheet_1.docx]
